# Preliminary insights of the genetic diversity and invasion pathways of *Cedrela odorata* in the Galapagos Islands, Ecuador

**DOI:** 10.1002/ece3.11723

**Published:** 2024-07-10

**Authors:** Martina Albuja‐Quintana, Gonzalo Rivas‐Torres, Karla E. Rojas López, Pacarina Asadobay, Walter Palacios Cuenca, Génesis Vinueza, Maria de Lourdes Torres

**Affiliations:** ^1^ Colegio de Ciencias Biológicas y Ambientales, Laboratorio de Biotecnología Vegetal Universidad San Francisco de Quito (USFQ) Quito Ecuador; ^2^ Colegio de Ciencias Biológicas y Ambientales Universidad San Francisco de Quito USFQ Quito Ecuador; ^3^ Galápagos Science Center Universidad San Francisco de Quito San Cristobal Ecuador; ^4^ University of North Carolina at Chapel Hill Chapel Hill North Carolina USA; ^5^ Estación de Biodiversidad Tiputini, Colegio de Ciencias Biológicas y Ambientales Universidad San Francisco de Quito USFQ Quito Ecuador; ^6^ Herbario Nacional del Ecuador (QCNE)‐Instituto Nacional de Biodiversidad Quito Ecuador

**Keywords:** *Cedrela odorata*, Galapagos Islands, genetic diversity, invasion history, invasive species

## Abstract

*Cedrela odorata* is considered the second most invasive tree species of the Galapagos Islands. Although it is listed in CITES Appendix II and there are population losses in mainland Ecuador, in Galapagos it is paradoxically a species of concern due to its invasive potential. Genetic studies can shed light on the invasion history of introduced species causing effects on unique ecosystems like the Galapagos. We analyzed nine microsatellite markers in *C. odorata* individuals from Galapagos and mainland Ecuador to describe the genetic diversity and population structure of *C. odorata* in the Galapagos and to explore the origin and invasion history of this species. The genetic diversity found for *C. odorata* in Galapagos (*H*_e_ = 0.55) was lower than reported in the mainland (*H*_e_ = 0.81), but higher than other invasive insular plant species, which could indicate multiple introductions. Our results suggest that Ecuador's northern Coastal region is the most likely origin of the Galapagos *C. odorata*, although further genomic studies, like Whole Genome Sequencing, Rad‐Seq, and/or Whole Genome SNP analyses, are needed to confirm this finding. Moreover, according to our proposed pathway scenarios, *C. odorata* was first introduced to San Cristobal and/or Santa Cruz from mainland Ecuador. After these initial introductions, *C. odorata* appears to have arrived to Isabela and Floreana from either San Cristobal or Santa Cruz. Here, we report the first genetic study of *C. odorata* in the Galapagos and the first attempt to unravel the invasion history of this species. The information obtained in this research could support management and control strategies to lessen the impact that *C. odorata* has on the islands' local flora and fauna.

## Graphical abstract

*Cedrela odorata* is the second most invasive tree species in the Galapagos Islands. Using nine microsatellite markers, we found that the genetic diversity of *C. odorata* in Galapagos is lower (*H*_e_ = 0.55) than in mainland Ecuador (*H*_e_ = 0.81). Our preliminary findings suggest multiple introduction events and that the origin of *C. odorata* in Galapagos could have been the north coast of mainland Ecuador. Possibly, it first reached San Cristobal or Santa Cruz and then extended to Isabela and Floreana, information that provides insights for its management.

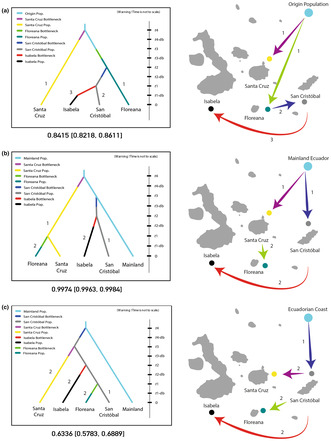

## Resumen

*Cedrela odorata* es considerada la segunda especie más invasora de árboles en las Islas Galápagos. Esta especie está catalogada en el Apéndice II de CITES y sus poblaciones se encuentran amenazadas en Ecuador continental, pero paradójicamente en Galápagos es una especie de preocupación por su potencial invasor. Estudios genéticos pueden ayudar a entender la historia de invasión de especies introducidas que causan efectos en ecosistemas únicos como Galápagos. En este estudio, analizamos 9 marcadores microsatélites en individuos de Galápagos y Ecuador continental para describir la diversidad genética y estructura poblacional de *C. odorata* en Galápagos y explorar el origen e historia de invasión de esta especie. La diversidad genética encontrada para *C. odorata* en Galápagos (*H*_e_ = 0.55) fue menor que la reportada en continente (*H*_e_ = 0.81), pero mayor que la de otras especies de plantas insulares invasoras, lo que podría sugerir múltiples introducciones de esta especie a Galápagos. Nuestros resultados sugieren que la costa norte ecuatoriana es el origen más probable de *C. odorata* en Galápagos, aunque más estudios, como secuenciación del genoma completo, Rad‐Seq y/o análisis de SNPs, son necesarios para confirmar este hecho. Además, de acuerdo con los escenarios propuestos, es posible que *C. odorata* haya sido introducida primero a San Cristóbal y/o Santa Cruz desde Ecuador continental. Después de estas introducciones iniciales, parece haber llegado a Isabela y Floreana desde San Cristóbal o Santa Cruz. Este es el primer estudio genético de *C. odorata* en Galápagos y el primer intento de esclarecer la historia de invasión de esta especie. La información obtenida en esta investigación podría apoyar estrategias de manejo para disminuir el impacto que *C. odorata* tiene sobre la flora y fauna nativa de estas islas.

## 1 INTRODUCTION

Invasive alien species usually experience rapid genetic changes due to selective pressures that allow them to persist, spread, and dominate their introduced range (Bossdorf et al., 2005; Hierro et al., 2005; Lawson Handley et al., 2011; Sakai et al., 2001), causing significant negative impacts on the biodiversity, ecosystem function, human welfare, and economy of recipient communities (Diagne et al., 2021). In general, successful colonization of invasive species can usually be attributed to ecological (e.g., high dispersal rates) or environmental (e.g., low number of competitors) factors (Sakai et al., 2001), but molecular focused studies suggest that genetic variability also plays a vital role to explain effective introductions (Lee & Gelembiuk, 2008).

Introduction events are characterized by changes in population allele frequencies caused by founder effects and strong bottleneck events (Kolbe et al., 2004; Lawson Handley et al., 2011). Theory predicts that rapid and successful invasions are usually aided by high genetic diversity that allows alien species to establish and quickly adapt to their new environment (Le Roux et al., 2008). Although most founder populations usually introduce only a small portion of the gene pool of their population of origin (Dlugosch & Parker, 2008; Sakai et al., 2001), many successful invaders maintain substantial levels of genetic diversity due to the occurrence of multiple introduction events (Kolbe et al., 2004; Lawson Handley et al., 2011). For instance, a study found that the invasive brown anoles of Florida likely experienced eight different introduction events that elevated their genetic diversity even above the values observed in their native range (Kolbe et al., 2004).

Population genetic studies can help elucidate possible sources of introduction of alien species, invasion routes, long‐term ecological and evolutionary changes (Le Roux & Wieczorek, 2008), and the genetic factors behind the ability of a species to adapt to new environments and become invasive (Torres & Gutiérrez, 2018). Additionally, and very importantly, the study of the genetic diversity of an invasive species can help establish the occurrence of single or multiple introduction events, genetic bottleneck events, and the source of introduction of a species by analyzing shared genetic variation between donor and recipient populations (Chaves, 2018; Torres et al., 2023).

Understanding the invasion processes of alien invasive species is especially important in insular ecosystems like the Galapagos. Islands tend to have lower species richness, simpler trophic assemblages, and higher habitat availability, making them intrinsically more sensitive to biological invasions (Stachowicz & Tilman, 2005; Vitousek, 1990). Growing anthropogenic intervention and agriculture in these island ecosystems negatively affects the native vegetation and soil, further allowing invasive species to settle, adapt, disperse, and dominate such environments (Mauchamp & Atkinson, 2010).

The Galapagos Islands, known for high endemism and ecologically specialized organisms (Tye et al., 2002), have now more than ~800 introduced plant species, 15% of which are considered invasive (Rivas‐Torres et al., 2017). Plant species have been mainly introduced to the Galapagos Islands as ornamental plants (e.g., *Lantana camara*), sources of timber (e.g., *Cedrela odorata*), agriculture (e.g., *Psidium guajava*, *Rubus niveus*), and for medicinal purposes (e.g., *Cinchona pubescens*) (Buddenhagen et al., 2004; Quinton et al., 2011; Rivas‐Torres et al., 2017; Urquía et al., 2021); nonetheless, not all of them have become invasive.

The invasion history and genetic characterization of Galapagos invasive plant species such as *Rubus niveus* (Quinton et al., 2011), *Psidium guajava* (Urquía et al., 2021), and *Solanum pimpinellifolium* (Gibson et al., 2021) have been studied, and depending on the case, low genetic diversity, introgression with endemic species, and introduction to the Galapagos from mainland Ecuador have been found. However, there are still several problematic invasive species that remain unexplored.

*Cedrela odorata* L. (Meliaceae) is a highly invasive tree species of the Galapagos Islands that is considered a tropical timber species of global economic importance (Cintron, 1990). *C. odorata* is possibly the most popular timber species of the genus *Cedrela*, which includes around 18 extant described species, but many more are currently being described due to *C. odorata*'s cryptic nature (Cavers et al., 2003; Palacios et al., 2023; Pennington & Muellner, 2010). Like many *Cedrela* species, *C. odorata* is a semideciduous, fast‐growing tree with a canopy reaching up to 30 m tall and producing thousands of wind‐dispersed seeds (James et al., 1998). Its distribution ranges from Mexico along Central America and the Caribbean up to Northern Argentina (Cavers et al., 2013).

In mainland Ecuador, *C. odorata* is classified as a vulnerable species by the IUCN (2017) and listed under CITES Appendix II (2007) due to the heavy exploitation of its timber, but in the Galapagos Islands it is considered the second most invasive tree species. Nowadays, this tree has spread over the four inhabited islands of the Galapagos (Santa Cruz, San Cristobal, Isabela, and Floreana) covering both agricultural and protected areas that were previously occupied by endemic and native species (Rivas‐Torres & Rivas, 2018). *C. odorata* dominates forests that were previously covered by *Scalesia*, an endemic species whose population has been reduced to 1% of its original extent since the arrival of *C. odorata* (Laso et al., 2020; Mauchamp & Atkinson, 2010; Rivas‐Torres & Rivas, 2018). *C. odorata* not only changes indigenous community composition but reduces native and endemic plant diversity in the islands through mechanisms already described, such as allelopathy, where *C. odorata* releases chemical compounds to inhibit the germination or growth of native and endemic species of the islands (Rivas‐Torres et al., 2017; Rivas‐Torres & Rivas, 2018).

**FIGURE 1:**
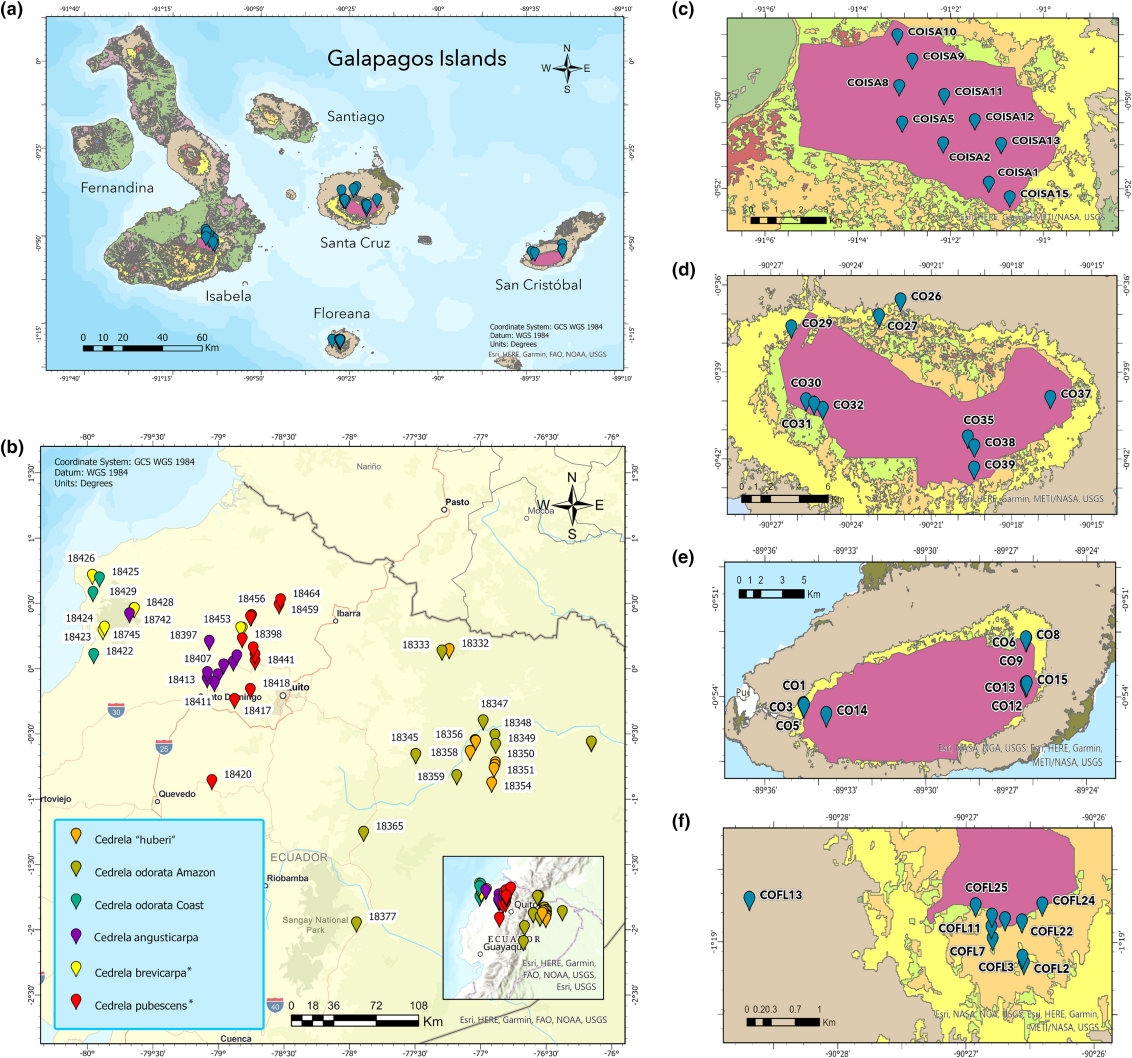
Sampling locations of *C. odorata* in the Galapagos and mainland Ecuador. For Sample IDs, see Appendix 2: Table A1. (a) Sampling sites of 40 individuals of *C. odorata* in the Galapagos that were collected in (c) Isabela, (d) Santa Cruz, (e) San Cristobal, and (f) Floreana. (b) Sampling sites of 68 individuals of *Cedrela* spp. collected in mainland Ecuador.

According to available historical records, *C. odorata* was introduced to Santa Cruz in the Galapagos Islands around 1940 as a source of timber, mainly for shipbuilding and furniture (Lundh, 2006). Nowadays, the largest population of this tree (~1000 hectares) can be found in the southwestern region of Santa Cruz (Figure 1d) where it spreads over agricultural areas and the edges of the Galapagos National Park (Rivas‐Torres & Rivas, 2018).

The paradox between *C. odorata*'s vulnerable status in the mainland and its invasive status in the Galapagos has made a complex task of legislative decisions surrounding the management and control of this invasive tree species. Harvesting *C. odorata* in the islands was even prohibited in 2007 due to its vulnerable status on the mainland (Rivas‐Torres & Rivas, 2018). This is further aggravated by the socio‐political interests surrounding the value of this tree for the archipelago's population (Rentería & Buddenhagen, 2006). Currently, only manual methods of control are being employed. Seedlings are uprooted by hand and controlled by chemicals while adults are chopped down (Charles Darwin Foundation, 2023). Despite the negative effects and significance of this tree in the Galapagos, little is known about the origin and genetic variation of *C. odorata*, therefore it is relevant to generate baseline data for this invasive species' control.

The aim of this research was to provide preliminary evidence about the invasion history of *Cedrela odorata* in the Galapagos using microsatellite markers. We describe the genetic diversity, population structure, and connectivity of *C. odorata* in the four inhabited islands of the Galapagos. We also explore the possible source of introduction and routes of invasion of this tree in the archipelago. The results of this research could contribute to define management strategies to lessen the impact of *C. odorata* on the islands' local flora and fauna.

## 2 MATERIALS AND METHODS

### 2.1 Sampling

Between February and March of 2018, leaves were collected from 40 individuals of *C. odorata* on the four inhabited islands: Floreana, Santa Cruz, San Cristobal, and Isabela (10 individuals from each island). To have a representative collection of the distribution range of *C. odorata* in the Galapagos, samples were collected in the lowlands, highlands, and edges of distribution of each of the four islands studied, both in agricultural fields and in protected areas (Figure 1a,c–f, Appendix 2: Table A1). Samples were collected at a minimum distance of 20 m from each other following suggested sampling distances from previous studies (Augspurger, 1983; Flesch et al., 2019).

To determine the possible origin and identity of the invasive *C. odorata*, 68 samples were collected from seven provinces along the native Coastal, Andean, and Amazon regions of Ecuador between May 2018 and March 2021 (Figure 1b, Appendix 2: Table A1). After a more detailed examination, some of the collected samples were reclassified as different *Cedrela* species by Walter Palacios Cuenca (Ecuadorian Meliaceae taxonomist). The samples corresponding to other *Cedrela* species were included in the UPGMA phylogenetic tree (see below) but excluded from other analyses.

Two to four leaves were collected from each individual tree and placed in sealed bags with silica gel for transport to the Plant Biotechnology Laboratory at Universidad San Francisco de Quito for genetic analyses. All collected samples have an herbarium voucher stored at QUSF Herbarium for comparative and taxonomic purposes.

### 2.2 DNA extraction and microsatellite genotyping

DNA was extracted from 20 mg of silica‐dried leaf tissue following the protocol for recalcitrant plants described by Rezadoost et al. (2016). The quality, quantity, and integrity of extracted DNA were assessed using a NanoDrop 2000 (Thermo Fisher Scientific) and an agarose gel (1%) electrophoresis.

Nine homologous microsatellite markers (SSR) developed for *C. odorata* by Hernández et al. (2008) were used for polymerase chain reaction (PCR) amplification. The nine SSR regions were amplified with fluorophore‐labeled forward primers (Appendix 2: Table A2). The PCR master mix had a total volume of 30 μL containing Ultra‐Pure PCR Water, 1X PCR Buffer, 2.5 mM MgCl_2_, 0.2 mM dNTPs, 0.2 μM forward and reverse primers, 1 U per reaction of Invitrogen© Platinum Taq DNA Polymerase, 0.016 mg/mL BSA, and 100 ng/μL of DNA per sample. To ensure amplification success, the final concentrations of BSA and MgCl_2_ were modified for certain primers and samples (Appendix 2: Table A3).

The polymerase chain reactions (PCR) were performed using the thermocycler program defined by Hernández et al. (2008) with an initial denaturation of 1 min at 94°C followed by 30 to 40 cycles [depending on the primer] of denaturation for 1 min at 94°C, annealing for 1 min at 55°C, extension for 1 min at 72°C, and a final extension of 5 min at 72°C. Some variations were made for annealing temperatures for the amplification success of six primers of some Galapagos samples (Appendix 2: Table A3). PCR products were visualized on agarose gel (1.5%) electrophoresis (35 min, 100 V) and then transferred to MicroAmp™ Optical 96‐Well Reaction Plates and sent off to Macrogen South Korea for genotyping. Genotypes were determined through capillary electrophoresis in an ABI 3130 Genetic Analyzer (ThermoFisher Scientific, USA) using 500LIZ as size standard. Results were analyzed in GeneMarker® software (SoftGenetics LLC 2012).

### 2.3 Statistical analyses

#### 2.3.1 Genetic diversity of *C. odorata* in the Galapagos Islands

Number of alleles (*N*_a_), expected (*H*_e_), and observed heterozygosity (*H*_o_) per locus were estimated on Rstudio v.4.0.3 with the *summary(genind)* function of the adegenet v. 2.1.3 package (Jombart et al., 2020). Deviations from Hardy–Weinberg equilibrium were calculated for each locus on Genepop v. 4.7 (Raymond & Rousset, 1995). The probability test was performed under the default Markov Chain parameters (Dememorization number: 1000, Number of batches: 100, Number of iterations per batch: 1000). Furthermore, FreeNa (Chapuis & Estoup, 2007) was used to estimate null allele frequency (*N*_o_) per locus, considering the expectation maximization (EM) algorithm. Given the fact that null alleles can bias population structure analysis and genetic diversity statistics (Dakin & Avise, 2004), corrected *F*_ST_ values were compared to uncorrected values using the excluding null alleles (ENA) method with a paired *t*‐test (*α* = .05).

Furthermore, genetic diversity indicators were estimated on Rstudio v. 4.0.3 for the four sampled islands in the Galapagos. Number of alleles (*N*_a_) were calculated with the *summary(genind)* function and expected heterozygosity (*H*_e_) with the *H*_s_ function of the adegenet v. 2.1.3 package (Jombart et al., 2020) while observed heterozygosity (*H*_o_) and inbreeding coefficients (*F*_IS_) were estimated with the *basic.stats* function of the hierfstat v. 0.5‐7 package (Goudet et al., 2020). The *private_alleles* function of the poppr v. 2.9.0 package (Kamvar et al., 2021) was used to estimate the number of private alleles (*N*_pa_) per island. Allelic richness (*R*_s_) per island was calculated using the *allel.rich* function of the PopGenReport v. 3.0.4 package (Gruber & Adamack, 2019).

#### 2.3.2 Genetic differentiation and population structure of *C. odorata* in the Galapagos Islands

An analysis of molecular variance (AMOVA) was conducted in Arlequin v. 3.5.2.2 (Weir & Cockerham, 1984) to evaluate the level of genetic differentiation found between and within the islands. The significance was estimated using 10,000 permutations. Pairwise fixation indexes (*F*_ST_) were also calculated in Rstudio v. 4.0.3 with the *pairwise.fstb* function of the PopGenReport v. 3.0.4 package (Gruber & Adamack, 2019). A Mantel test was performed (23 permutations: maximum number of permutations allowed with our data set) with the *mantel* function of the vegan v. 2.5‐7 package (Oksanen et al., 2020) to assess a possible correlation between genetic and geographic distances of the individual islands. In order to determine the directional gene flow and its relative magnitude among the islands analyzed in this study, the R‐function *divMigrate* (Sundqvist et al., 2016) implemented in the R‐package diveRsity v.1.9.9 (Keenan, 2017) was used.

To evaluate the population structure of *C. odorata* in the Galapagos, a discriminant analysis of principal components (DAPC) was performed with the *dapc* function of the adegenet v. 2.1.3 package (Jombart et al., 2020). DAPC gives higher priority to inter‐group than intra‐group variation detecting clusters without the assumptions of HW and linkage equilibrium (Jombart et al., 2020). The optimum number of genetic clusters was predicted using a *K*‐means clustering algorithm via the Bayesian information criterion (BIC). The most likely number of genetic clusters was associated with the lowest BIC values determined with the *find.clusters* function of the adegenet v. 2.1.3 package (Jombart et al., 2020). The final graph was plotted with the *scatter.dapc* function.

#### 2.3.3 Possible source of introduction and identity verification of *C. odorata* in the Galapagos Islands

To determine the possible origin of *C. odorata* in the Galapagos and verify its identity, a phylogenetic tree was constructed by UPGMA using Nei's genetic distance (adegenet v. 2.1.3 package, Jombart et al., 2020) in Rstudio v.4.0.3 with the *aboot* function of the poppr v. 2.9.0 package (Kamvar et al., 2021). Samples of *C. odorata* from the Galapagos (introduced range) and *Cedrela* species from mainland Ecuador (native range) were used, and 1000 bootstraps were run to analyze node support.

The analysis of the possible origin of invasive *C. odorata* in the Galapagos was further explored by analyzing samples from other regions of *C. odorata* native neotropical distribution. Microsatellite data for six loci (Ced44, Ced41, Ced61a, Ced65, Ced95, Ced131) of the nine used in this study were provided by Dr. Stephen Cavers for 528 individuals sampled along *C. odorata* native range (Cavers et al., 2013). A PcoA was performed with the *pcoa* function of the ape v. 5.4‐1 package (Paradis et al., 2020) and plotted with the ggplot function of the ggplot2 v. 3.3.3 package (Wickham et al., 2020). Data were grouped and divided into the following regions: North, Central, and South America, the Caribbean, Galapagos, Coastal, and Amazon regions in Ecuador (Appendix 2: Table A4).

#### 2.3.4 Predicting possible routes of invasion for *C. odorata* in the Galapagos Islands

To infer the possible routes of invasion of *C. odorata* in the Galapagos Islands, approximate Bayesian computation (ABC) analyses were performed on DIYABC (v2.1.0) (Cornuet et al., 2014). This software calculates posterior probabilities of proposed scenarios of introduction, comparing simulated and observed summary statistics of each scenario through logistic regressions (Cornuet et al., 2014). A total of 35 different scenarios were proposed and run along three stages. In the first stage, only individuals sampled in Galapagos were evaluated and the population of origin was specified as having an unknown effective population size (*N*_e_). For a more exhaustive analysis of the modeled scenarios, only the 15 most probable scenarios found in Stage 1 were analyzed in the second and third stages. In Stage 2, samples of *C. odorata* from mainland Ecuador were added to the samples from Galapagos. The samples from the Coastal and Amazon regions were specified as the population of origin. In Stage 3, samples of *C. odorata* from the Coastal region were specified as the population of origin and were analyzed together with the samples from Galapagos.

Scenarios were built based on literature review (Astudillo, 2018; Gordillo, 2000; Lundh, 2006) and previous experience, testing scenarios with one to multiple independent introductions to the various islands. Bottleneck events were considered after each introduction, and admixture origins were also tested in multiple scenarios for the four islands. In total, 1,000,000 simulations were run for each scenario following a stepwise mutation model (SMM) and using mean number of alleles, mean genic diversity, and *F*_ST_ values as summary statistics. All scenarios were plotted considering effective population sizes for the four islands and mainland origin (Ne/1, *N*2, *N*3, *N*4, *N*5), post–bottleneck population sizes (*N*2b, *N*3b, *N*4b, *N*5b), times of divergence between populations (*t*1, *t*2, *t*3, *t*4), and duration of bottleneck events (*t*1‐db, *t*2‐db, *t*3‐db, *t*4‐db). A logistic regression estimate was used to calculate the posterior probabilities of each scenario, and demographic parameters were calculated for each scenario using the “estimate parameters” function of DIYABC (v2.1.0) (Cornuet et al., 2014). Additionally, the goodness of fit of the best‐supported scenarios were also evaluated by principal component analyses (PCA) using the option “model checking” in DIYABC and the type I and mean type II errors were calculated running simulations of all scenarios with the “confidence in scenario choice” option.

## 3 RESULTS

### 3.1 Genetic diversity of *C. odorata* in the Galapagos Islands

All nine homologous microsatellites tested were polymorphic with an average of 3.9 alleles per locus, ranging from 2 to 7 alleles. The number of alleles (*N*_a_) found for each island ranged from 21 (Isabela) to 29 (Santa Cruz). Private alleles represented 11% of all reported alleles, varying from 1 in Isabela to 5 in Santa Cruz. Null allele frequency ranged up to 0.28 (Ced18), but no significant differences were found between uncorrected and corrected *F*_ST_ values (*p* = .47, *t* = 0.068). Three loci (Ced131, Ced65, and Ced54) of the nine analyzed showed significant deviations from Hardy–Weinberg equilibrium but were not excluded from the analyses (Appendix 2: Table A5).

Observed heterozygosity (*H*_o_) per locus varied from 0.07 (Ced18) to 0.65 (Ced61a) while expected heterozygosity (*H*_e_) per locus ranged from 0.32 (Ced131) to 0.74 (Ced95/Ced61a), making Ced95 and Ced61a the most informative SSRs (Appendix 2: Table A5). Expected heterozygosity (*H*_e_) was higher than observed heterozygosity (*H*_o_) in all four islands. Observed heterozygosity (*H*_o_) ranged from 0.23 (San Cristobal) to 0.53 (Santa Cruz), with a global value of 0.36. Expected heterozygosity (*H*_e_) ranged from 0.35 (San Cristobal) to 0.55 (Santa Cruz); with a global value of 0.55 (Appendix 1: Table A6).

The population of Santa Cruz presented the highest genetic diversity values (*H*_e_ = 0.55, *H*_o_ = 0.53, *R*_s_ = 3.15) while San Cristobal presented the lowest (*H*_e_ = 0.35, *H*_o_ = 0.23, *R*_s_ = 2.32). Moreover, the degree of inbreeding (*F*_IS_) was moderately low (mean = 0.23), ranging from 0.07 in Santa Cruz to 0.36 in San Cristobal (Appendix 2: Table A6).

### 3.2 Genetic differentiation and population structure of *C. odorata* in the Galapagos Islands

The analysis of molecular variance (AMOVA) demonstrated that 20.70% of the reported variation was observed between populations and 79.30% within the analyzed island populations. The reported pairwise *F*_ST_ distances (*F*_ST_ = 0.079–0.191) (Table 1) suggested certain degree of differentiation among the islands. Isabela and San Cristobal, the furthest islands from each other, presented the lowest genetic differentiation (*F*_ST_ = 0.079). Interestingly, this low degree of differentiation did not coincide with the geographic distances between the islands since no significant correlation was found between genetic and geographical distances of the islands in the Mantel test performed (*p* = .833, *r* ^2^ = −.37).

**TABLE 1:** Pairwise *F*_ST_ distances between the islands where *C. odorata* is distributed in the Galapagos Islands.

	Santa Cruz	San Cristobal	Isabela	Floreana
Santa Cruz	—	0.134	0.119	0.126
San Cristobal	0.134	—	0.079	0.185
Isabela	0.119	0.079	—	0.191
Floreana	0.126	0.185	0.191	—

*Note*: It is suggested that *F*_ST_ values between 0 and 0.05 represent little genetic differentiation, 0.05–0.15 moderate genetic differentiation, 0.15–0.25 great genetic differentiation, and >0.25 very great genetic differentiation (Hartl & Clark, 1997).

**FIGURE A1:**
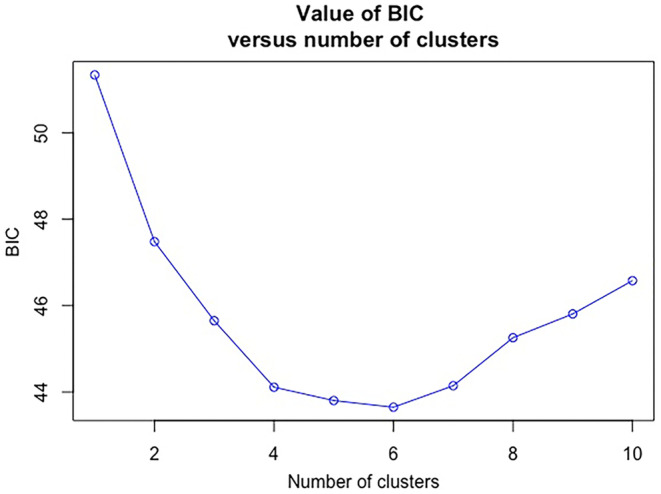
Population genetic clustering results based on the Bayesian Information Criterion (BIC) in relation to the number of clusters identified by the *find.cluster* function in DAPC analysis. Six distinct genetic clusters were identified.

The DAPC clustering algorithm based on BIC suggested six distinct clusters for the *C. odorata* of Galapagos (Appendix 1: Figure A1). The main axes of the DAPC analysis clearly separated samples of Floreana (represented by two clusters) from the other islands.

**FIGURE 2:**
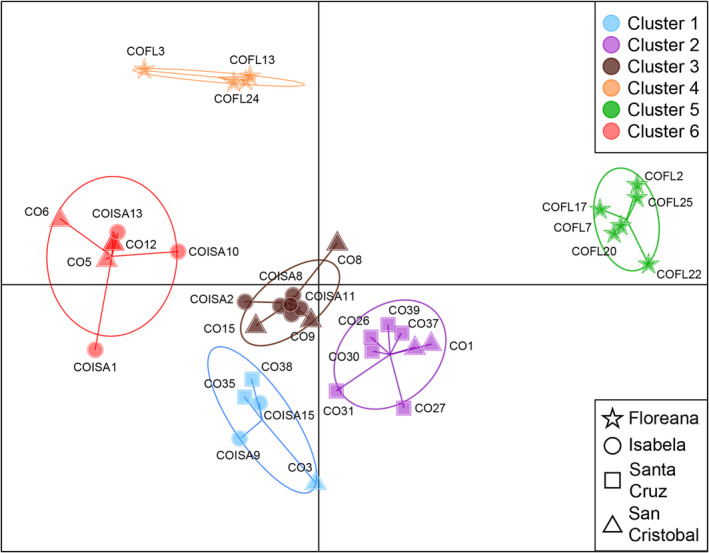
Discriminant analysis of principal components (DAPC) of the *C. odorata* of Galapagos grouped into six clusters on the first two axes of DAPC. Each point represents an individual, where color signifies the assignment of individuals to clusters, while shape denotes their population of origin.

Samples from Santa Cruz were found mainly on Cluster 2 (with some samples from San Cristobal) but also on Cluster 1 were samples from all islands except Floreana converged. The other two clusters were shared by individuals from Isabela and San Cristobal which could demonstrate a greater genetic proximity between samples from these two islands. The presence of six different genetic clusters suggests the possibility of multiple introduction events and a clear separation of the samples of Floreana from the other islands (Figure 2).

**FIGURE A2:**
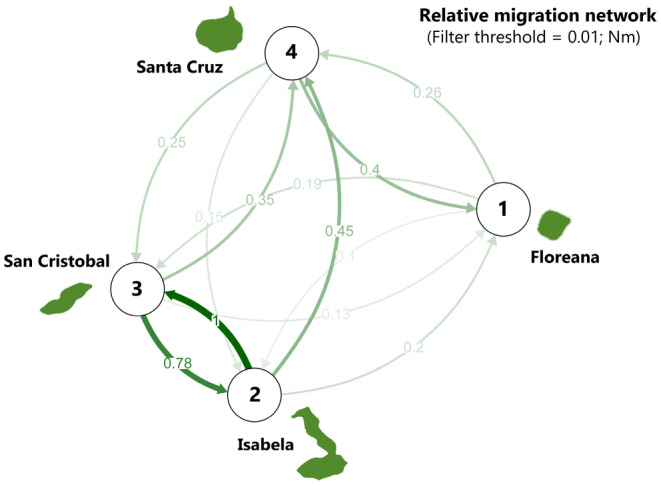
Directional gene flow and relative migration network among the four islands of the Galapagos where *C. odorata* is distributed. The distance between the points is proportional to the genetic differentiation between the islands while the arrows indicate the directionality, and the numbers indicate the rate of gene flow (0–1). Color shade and line thickness are proportional to the relative strength of gene flow (Sundqvist et al., 2016).

The relative migration network (Appendix 1: Figure A2) revealed a bidirectional symmetric gene flow between all islands. The highest migration rates (0.78–1) were reported between San Cristobal and Isabela, while the lowest were reported between Isabela and Floreana (0.1–0.2) and between San Cristobal and Floreana (0.13–0.19). Moderate migration rates (0.15–0.45) were observed between Santa Cruz and the other islands.

### 3.3 Possible source of introduction and identity verification of *C. odorata* in the Galapagos Islands

**FIGURE 3:**
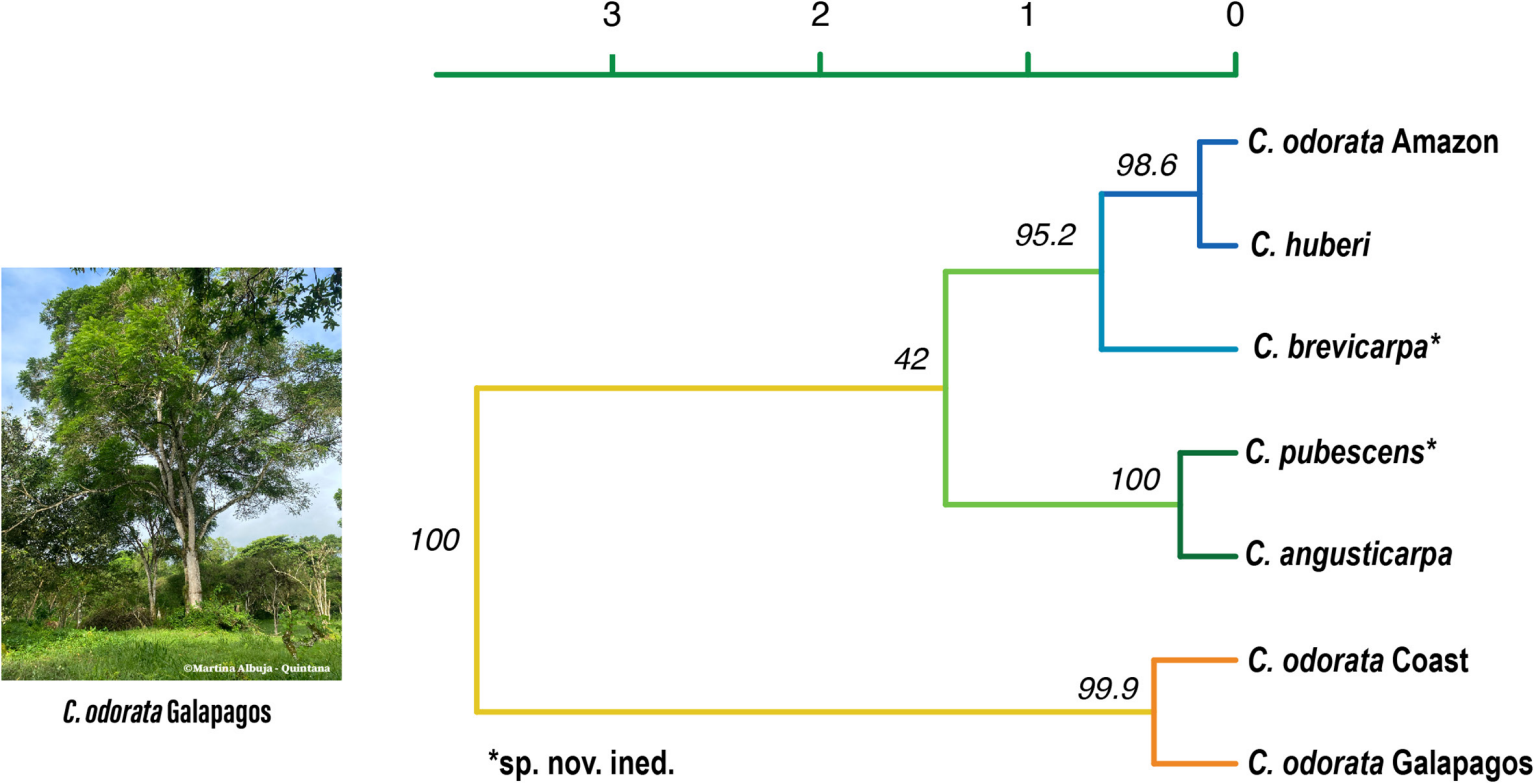
UPGMA dendrogram of genetic relationships between four native *Cedrela* species from mainland Ecuador and three populations of *C. odorata* using Nei's genetic distance based on nine microsatellite loci. Values specified in each node represent bootstrap values in % (1000 bootstraps).

The UPGMA phylogenetic tree, based on Nei's genetic distances (1000 bootstraps), showed that the *C. odorata* from the Galapagos clustered close to the *C. odorata* from the Coast of Ecuador and were largely differentiated (bootstrap value: 99.9%) from other *Cedrela* species and other Ecuadorian native populations of *C. odorata*. The branch of the tree where the Galapagos *C. odorata* samples are grouped argues that *C. odorata* is the species present in the Galapagos populations and suggests that the Ecuadorian Coast would be the likely source of introduction of this invasive species (Figure 3).

**FIGURE 4:**
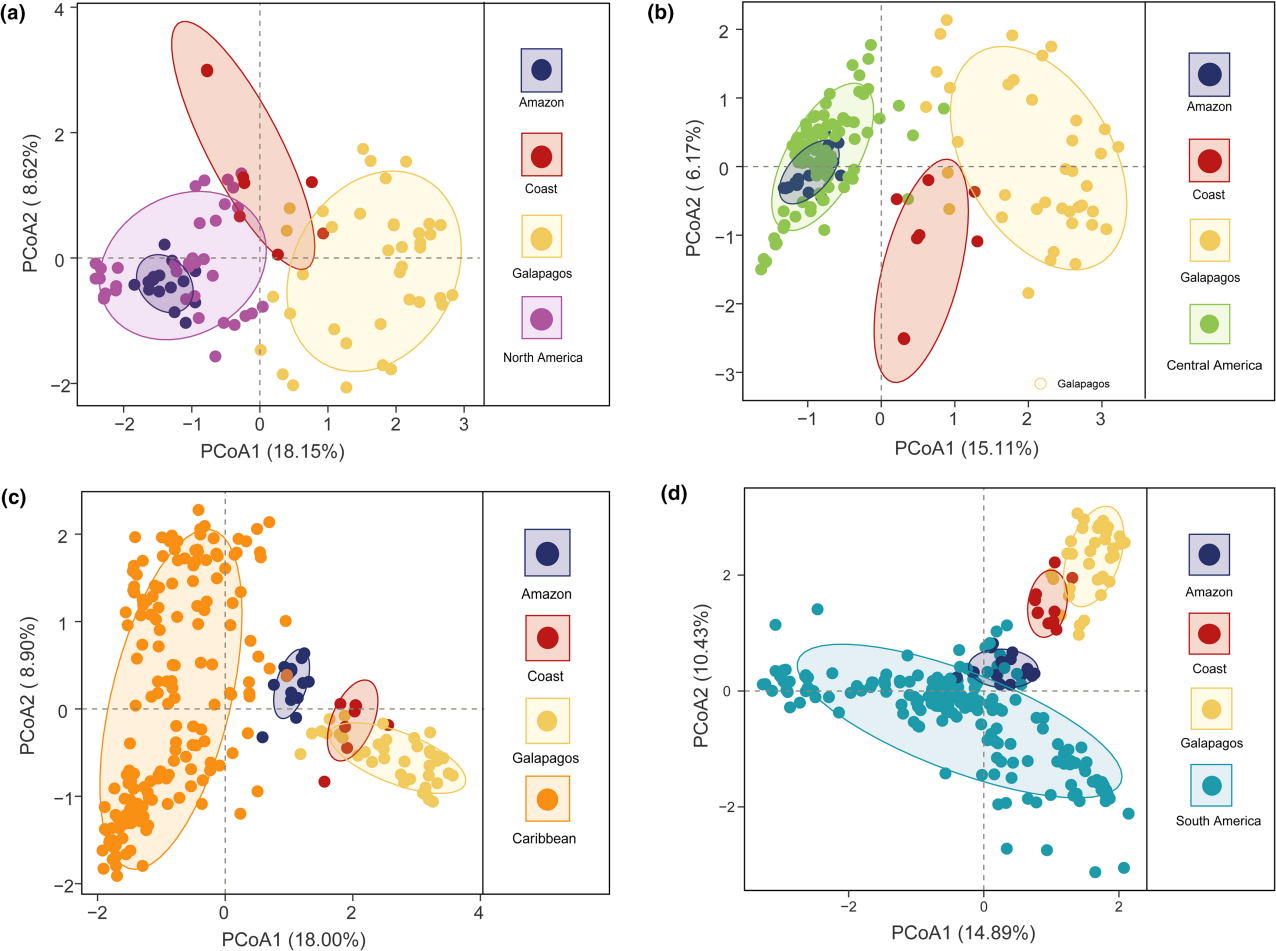
PcoA based on genetic distances using 6 SSR markers found for samples of *C. odorata* in Galapagos (yellow), Ecuadorian Amazon (dark blue), Ecuadorian Coast (dark red), North America (a: Purple), Central America (b: Green), The Caribbean (c: Orange) and South America (d: Turquoise) (Appendix 2: Table A4). The first two principal coordinates were considered.

The possible invasion origin (Coast of Ecuador) of *C. odorata* from Galapagos was corroborated when samples from other regions of *C. odorata*'s native range were analyzed (Figure 4). Galapagos *C. odorata* samples did not show a clear genetic association with any of the new regions (North America‐purple, South America‐turquoise, Central America‐green, and Caribbean‐orange), but clustered close to *C. odorata* from the Coastal region of Ecuador.

### 3.4 Predicting possible routes of invasion for *C. odorata* in the Galapagos Islands

**FIGURE 5:**
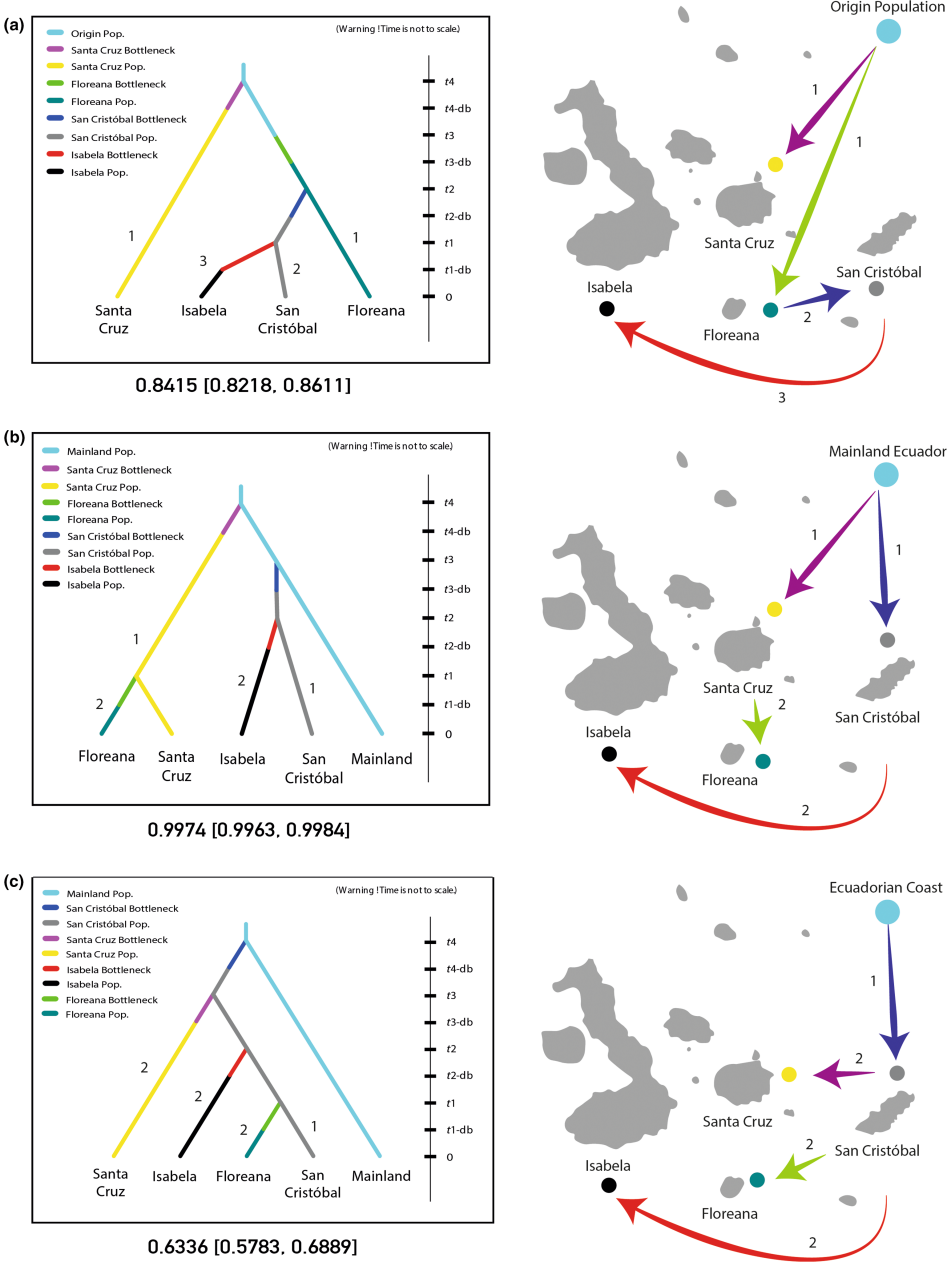
Possible routes of invasion of *C. odorata* in the Galapagos Islands estimated through ABC analyses. The best‐supported scenarios for the three stages are presented. (a) Diagram and map illustrating the best‐supported scenario of Stage 1 (0.8415). This scenario considers only samples from Galapagos and suggests initial introductions from mainland Ecuador to Floreana and Santa Cruz with subsequent introductions to San Cristobal from Floreana and from San Cristobal to Isabela. (b) Diagram and map illustrating the best‐supported scenario of Stage 2 (0.9974). This scenario considers samples from mainland Ecuador as the population of origin and proposes initial introductions from mainland Ecuador to Santa Cruz and San Cristobal, with subsequent introductions to Isabela from San Cristobal and to Floreana from Santa Cruz. (c) Diagram and map illustrating the best‐supported scenario of Stage 3 (0.6336). This scenario considers samples from the Coastal region as the population of origin and suggests an initial introduction from the Coastal region to San Cristobal with subsequent introductions to the other three islands.

Three best‐supported scenarios were obtained along the three stages of the ABC analyses (Figure 5). All three scenarios suggest that *C. odorata* in the Galapagos was first introduced to San Cristobal and/or Santa Cruz from mainland Ecuador. After these first introductions, *C. odorata* probably arrived on Isabela and Floreana from San Cristobal or Santa Cruz. A detailed description of each scenario is given in Figure 5.

**FIGURE A3:**
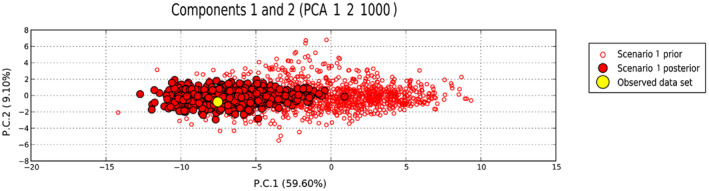
Model checking of the best supported scenario of Stage 1 (A) through a Principal Component Analysis (PCA).

**FIGURE A4:**
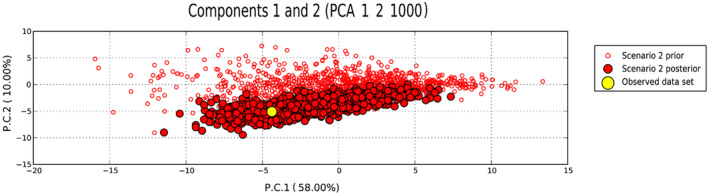
Model checking of the best supported scenario of Stage 2 (B) through a Principal Component Analysis (PCA). Observed and simulated (prior and posterior) datasets are compared.

**FIGURE A5:**
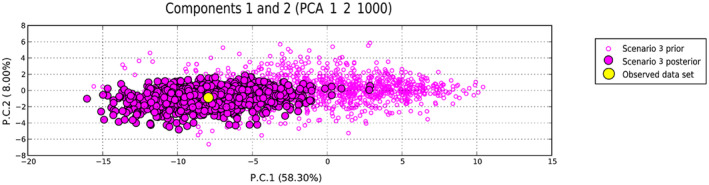
Model checking of the best supported scenario of Stage 3 (C) through a Principal Component Analysis (PCA).

The demographic parameters for all three scenarios including effective population sizes (Ne/1, *N*2, *N*3, *N*4, *N*5), times of divergence between populations (*t*1, *t*2, *t*3, *t*4), and microsatellite mutation rates (μmic) are summarized in Appendix 2: Table A7 (A), Table A8 (B), and Table A9 (C). The type I and mean II error values for the best‐supported scenarios for Stage 1 (A) were 0.34 and 0.06 [0.02–0.13] while for Stage 2 (B) and Stage 3 (C) were 0.29 and 0.08 [0.03–0.16], 0.23 and 0.08 [0.03–0.12], respectively. The relative median absolute error values were moderate in the parameter estimates for all scenarios (Appendix 2: Tables A7, A8, A9). The model checking in DIYABC was evaluated by principal component analyses (PCA), based on 10,000 simulations showing the fit between the observed and the simulated datasets (Appendix 1: Figures A3, A4, A5).

It is possible that *C. odorata* was introduced independently at least once or twice to the islands from mainland Ecuador, as two of the three scenarios coincide in that the introduction of this tree from the mainland did not occur on a single island.

## 4 DISCUSSION

### 4.1 Genetic diversity of *C. odorata* in the Galapagos Islands

Invasive species often start their colonization with the introduction of a small number of individuals to a new environment different from their native range. These founder individuals carry the genetic composition that will eventually be passed on to future generations (Chaves, 2018). As a result of founder and bottleneck events, invasive populations often show lower genetic diversity compared to their native range (Lawson Handley et al., 2011). This is the case for *C. odorata* populations in the Galapagos Islands (*H*_e_ = 0.55) when compared to mainland Ecuador (*H*_e_ = 0.81), Mesoamerica (*H*_e_ = 0.86), and Bolivia (*H*_e_ = 0.83–0.89), native *C. odorata* regions that have reported higher genetic diversity (Asadobay, 2019; Hernández, 2008; Paredes‐Villanueva et al., 2019).

Nonetheless, the genetic diversity of *C. odorata* found in the Galapagos is moderately high when compared to other invasive tree species of islands like *Psidium guajava* (Myrtaceae; *H*_e_ = 0.36) in Galapagos (Urquía et al., 2019) and *Clidemia hirta* (Melastomataceae; *H*_e_ = 0.06) in Hawaii (DeWalt & Hamrick, 2004). Moderately high comparable levels of genetic diversity have been documented in other invasive tree species such as *Acacia saligna* in South Africa, Australia, and Portugal (Thompson et al., 2015), *Paraserianthes lophantha subspecies lophantha* in Hawaii (Thompson et al., 2016), and *Acacia dealbata* in New Zealand (Hirsch et al., 2021). The recurrence of these genetic diversity values across multiple invasive tree species suggests that intrinsic biological factors could play a role in the observed genetic diversity. These factors include dispersal capabilities, mating systems, and generation times, which could collectively contribute to the successful invasion of these species (Stuessy et al., 2014). Several of these studies also propose the occurrence of multiple introduction events as a possible factor explaining the genetic diversity found. The genetic diversity found in *C. odorata* in the Galapagos may be explained by these intrinsic biological factors and by the possibility of several introductions that could have increased and modified the genetic composition of the initial populations that arrived in the Galapagos, allowing them to overcome founder effects and bottlenecks, and adapt successfully to their new environment (Kelly et al., 2006; Lawson Handley et al., 2011). This same phenomenon has been observed in other invasive species of the Galapagos Islands, such as mosquitoes (*Aedes aegypti*) in Santa Cruz (Chaves, 2018), and *Solanum pimpinellifolium* around San Cristobal and Santa Cruz (Torres et al., 2023).

*C. odorata* in the Galapagos Islands shows moderate levels of inbreeding. Within invasive populations, inbreeding is expected since founder effects and bottleneck events usually lead to nonrandom mating (Le Roux et al., 2010). Small founder populations or excessive self‐crossing increases the likelihood of mating between closely related individuals (Le Roux et al., 2010). *Cedrela* species are generally known for their high self‐incompatibility and predominantly outcrossing reproduction (Aschero, 2006; James et al., 1998). In a study conducted in Central America, no self‐crossing was observed between individuals of *C. odorata* found within 500 meters of each other (Hernández, 2008), further supporting the prevalence of outcrossing. The levels of inbreeding observed in Galapagos *C. odorata* populations would then likely be the result of founder effects.

### 4.2 Genetic differentiation and population structure of *C. odorata* in the Galapagos Islands

Our results show that there is a low genetic differentiation between *C. odorata* populations of the different islands (Table 1). This could be explained by *C. odorata* ecology and breeding patterns. *Cedrela* species, in general, tend to exhibit higher genetic variation within populations (Huamán, 2014; Mangaravite et al., 2019). This has been observed in other outcrossing, long‐lived woody plant species that usually maintain higher intrapopulation than interpopulation variation (Hamrick et al., 1992; Hamrick & Godt, 1996).

Alternatively, low genetic differentiation could be the result of constant gene flow between the islands. This dispersal is probably human‐mediated since *C. odorata* is considered the main source of timber for the inhabitants of the Galapagos (Rivas‐Torres & Adams, 2018). Although *C. odorata* pollinators are known to travel over long distances, only trees found up to 500 m apart have been seen to intercross (Hernández, 2008). The maximum flight distance recorded for *C. odorata* seeds is up to 100 m (Navarro, 2002), supporting the intervention of humans in its dispersal throughout the archipelago.

Despite the overall low genetic differentiation, some structure can be observed between the *C. odorata* populations of the different islands, with San Cristobal and Isabela being the most similar (Figure 2). This could be explained by historical patterns of trade and transportation between the islands. During the 1900s, most of the cargo leaving Isabela for the mainland was transported on vessels departing from San Cristobal, which was one of the main ports of the archipelago at the time (Lundh, 2001; Quiroga, 2013). These historical transport connections may have contributed to the genetic similarity observed between these two islands.

### 4.3 Possible source of introduction of *C. odorata* in the Galapagos Islands

Several invasive plant species of the Galapagos Islands are believed to have been introduced from mainland Ecuador. Species like *Psidium guajava* (Urquía et al., 2021) and *Solanum pimpinellifolium* (Gibson et al., 2021) probably were introduced from the Andean region in Central Ecuador. Likewise, our results suggest that the Coast of Ecuador may be the most likely source of introduction for *C. odorata* in the Galapagos.

The history of the Galapagos Islands can provide some clues for this coastal origin. In the 1900s, Guayaquil, the largest coastal city of Ecuador, was an important trading center between the Galapagos and mainland Ecuador (Toral‐Granda et al., 2017). Ships were the only means of transportation going into the archipelago (Gordillo, 2000). Most of these vessels traveled to and from Guayaquil (Vera, 1941), as this city was one of Ecuador's main ports, through which settlers, visitors, livestock, and goods passed to reach the islands (Lundh, 2001; Maldonado & Llerena, 2019; Toral‐Granda et al., 2017). Plant species that were cultivated in the agricultural regions surrounding Guayaquil were more easily traded on the islands (Gibson et al., 2021), which probably facilitated the introduction of useful plants from the Coast of Ecuador. Additionally, historical data report that Dr Pedro Holst, Danish consul in Guayaquil during the 1940s, was one of the persons who prompted the introduction of *C. odorata* seeds to the Galapagos (Lundh, 2006). Records even mention that Dr Holst sold and distributed *C. odorata* seeds throughout South America (Holst, 1960). If these records are accurate, *C. odorata* could have an average of 7 generations in the islands, considering that trees are known to start producing seeds at 12 years of age (Cintron, 1990). These seven generations have caused negative impacts such as allelopathy and species displacement as has been reported in other studies (Rentería & Buddenhagen, 2006; Rivas‐Torres & Rivas, 2018).

It is important to note that there are several reports of Cuban plantations of *C. odorata* near Guayaquil, which could potentially suggest a Central American origin for the Galapagos *C. odorata* (Cintron, 1990; Lamb, 1968; Wadsworth, 1960). Although our preliminary results show no genetic similarity between *C. odorata* in the Galapagos and Central America, this prediction needs to be explored further. The number of samples analyzed in Galapagos should be expanded and a wider geographical range of distribution should be covered to see if the results remain valid or if another geographical origin of introduction can be identified. If another source of introduction than the one reported in this study is found, it would probably contribute to a better understanding of the distribution of this invasive tree in the Galapagos. Currently, *C. odorata* is mainly found in the humid highlands of the islands, particularly near the agricultural area where it was first introduced (Rivas‐Torres & Adams, 2018). Nonetheless, it has now started to spread toward the drier areas of the islands, indicating its adaptability. *C. odorata* is considered a climate generalist, as it occurs in both dry and moist forest environments (Cintron, 1990). *C. odorata* lineages from Central America are typically associated with drier, seasonal conditions while lineages from South America tend to occupy areas exposed to higher levels of precipitation (Cavers et al., 2003, 2013). Knowing the origin(s) of an invasive species could provide crucial information on its success and dispersal throughout its introduced range. This could help to identify potential areas of the islands that could be infested in the future, which in turn could help to develop more effective management strategies. For instance, biological control strategies rely on host specificity to identify natural enemies capable of controlling invasive species in their introduced habitats (Prentis et al., 2009). Accurate host specificity is crucial to avoid unintended impacts on native or endemic plant species (Williams et al., 2005). Increasingly, research indicates that the most effective and host‐specific natural enemies are often from the same locality as the invasive species (Le Roux & Wieczorek, 2008; Roderick & Navajas, 2003). This entails not only identifying indigenous natural enemies, but also recognizing the potential influence of plant genotypes on host–enemy associations (Williams et al., 2005), emphasizing the need for genetic diversity studies to improve management strategies.

### 4.4 Predicting possible routes of invasion for *C. odorata* in the Galapagos Islands

According to our proposed scenarios (Figure 5), Santa Cruz seems to be one of the initial sites of introduction of the *C. odorata* in the Galapagos. *C. odorata* seems to have been introduced to the agricultural area of Santa Cruz in the 1940s and 1950s (Lundh, 2006; Tye, 2001). Santa Cruz served as one of the main ports in the Galapagos, acting as a point of contact between the mainland and the archipelago. Most vessels arriving from the mainland passed through Santa Cruz before heading to the other islands (Lundh, 2001). It is reasonable to believe that seeds of *C. odorata* may have first reached Santa Cruz before dispersing to the other inhabited islands of the Galapagos.

The invasion history of *C. odorata* in Santa Cruz is like another invasive tree species of the Galapagos Islands, the red quinine tree (*Cinchona pubescens*). The latter was also introduced to the agricultural area of Santa Cruz in 1946 and rapidly spread and colonized the island due to its numerous winged wind‐dispersed seeds (Buddenhagen et al., 2004). Both invasive tree species were introduced around the same time and quickly established themselves in Santa Cruz, taking advantage of their wind‐dispersed seeds and encountering little resistance and competition from endemic species like *Scalesia* (Rivas‐Torres et al., 2017).

However, unlike *C. pubescens*, *C. odorata* appears to have been also separately introduced to San Cristobal from the mainland (Figure 5). San Cristobal was also used as a point of entry into the archipelago, mainly due to its closer proximity to the mainland (Quiroga, 2013). Being the most populated island during the mid‐1900s (Lundh, 2001), San Cristobal frequently received people, letters, and goods from mainland Ecuador (Quiroga, 2013). It is possible that seeds of *C. odorata* were brought to this island on one of these trips. The possible dispersal route of *C. odorata* in the Galapagos Islands from San Cristobal is similar to that reported for *Rubus niveus* (Quinton et al., 2011) and *Psidium guajava* (Urquía et al., 2019), two species that were probably initially introduced in agricultural areas of San Cristobal, are naturalized, and are now found in other islands of the archipelago.

## 5 CONCLUSIONS

*Cedrela odorata* was introduced to the Galapagos because of its use by the colonizers of the islands. The efficient dispersal mechanism of *C. odorata*, the limited competition it faced with endemic and native species, and its adaptability to the local conditions allowed this tree to become an invasive species, and to spread and colonize large areas on the four inhabited islands of the archipelago. The moderately high genetic diversity and gene flow found in the *C. odorata* populations studied could have influenced its successful dispersal. The genetic diversity found could be due to multiple introduction events from mainland Ecuador. The Coast of Ecuador is proposed as a possible origin of *C. odorata* in the Galapagos, but further studies are required to confirm if this is the only source of introduction or if there are other possible sources. This study demonstrates that the combination of genetic analysis, historical records, and field observations are fundamental to reconstruct the invasion history of introduced species in island ecosystems, shedding light on the mechanisms driving the invasions (Torres et al., 2023). This research reports the first genetic study of *C. odorata* in the Galapagos Islands and the first attempt to unravel its invasion history. Our results are preliminary and should be strengthened with further studies covering more analyzed individuals and the use of genomic tools, like Whole Genome Sequencing, Rad‐Seq, and/or Whole Genome SNP analyses, that could give us more comprehensive information about the genetic status of this species. More information could contribute to reverse or avoid the effects that this species, and similar others, have on such a unique and fragile ecosystem as the Galapagos Islands.

## AUTHOR CONTRIBUTIONS

**Martina Albuja‐Quintana:** Data curation (equal); formal analysis (lead); investigation (equal); methodology (equal); software (equal); visualization (equal); writing – original draft (lead); writing – review and editing (equal). **Gonzalo Rivas‐Torres:** Conceptualization (lead); data curation (equal); funding acquisition (lead); investigation (equal); methodology (equal); project administration (lead); resources (equal); supervision (lead); validation (equal); writing – review and editing (equal). **Karla E. Rojas López:** Data curation (equal); formal analysis (supporting); investigation (equal); methodology (equal); software (lead); writing – review and editing (equal). **Pacarina Asadobay:** Data curation (equal); formal analysis (supporting); investigation (equal); methodology (equal); software (supporting). **Walter Palacios Cuenca:** Data curation (equal); investigation (equal); methodology (equal); resources (equal). **Génesis Vinueza:** Data curation (equal); formal analysis (supporting); investigation (equal); methodology (equal); software (supporting). **Maria de Lourdes Torres:** Conceptualization (lead); data curation (equal); formal analysis (equal); investigation (equal); methodology (equal); project administration (lead); resources (lead); supervision (lead); validation (lead); writing – review and editing (lead).

## CONFLICT OF INTEREST STATEMENT

The authors declare no competing or financial interests. The funders had no role in the design of the study, data collection, analyses, interpretation of data, manuscript writing, or in the decision to submit for publication.

## Data Availability

The dataset generated during and/or analyzed during the current study is available in the Zenodo repository, https://doi.org/10.5281/zenodo.8302098.

## APPENDIX 2

**TABLE A1:** Geographic information of *Cedrela spp.* samples collected in the Galapagos Islands and mainland Ecuador.

ID	Species	Locality	Latitude	Longitude	Collector	Altitude (m)	Voucher
CO26	*Cedrela odorata*	Galapagos Santa Cruz	−0.61532	−90.36913	G. Vinueza (2020)	571	QUSF
CO39	*Cedrela odorata*	Galapagos Santa Cruz	−0.71205	−90.32325	G. Vinueza (2020)	137	QUSF
CO29	*Cedrela odorata*	Galapagos Santa Cruz	−0.63088	−90.43728	G. Vinueza (2020)	394	QUSF
CO30	*Cedrela odorata*	Galapagos Santa Cruz	−0.67282	−90.42805	G. Vinueza (2020)	246	QUSF
CO37	*Cedrela odorata*	Galapagos Santa Cruz	−0.67140	−90.27580	G. Vinueza (2020)	270	QUSF
CO27	*Cedrela odorata*	Galapagos Santa Cruz	−0.62443	−90.38260	G. Vinueza (2020)	615	QUSF
CO32	*Cedrela odorata*	Galapagos Santa Cruz	−0.67777	−90.41762	G. Vinueza (2020)	233	QUSF
CO31	*Cedrela odorata*	Galapagos Santa Cruz	−0.67482	−90.42302	G. Vinueza (2020)	250	QUSF
CO35	*Cedrela odorata*	Galapagos Santa Cruz	−0.69412	−90.32717	G. Vinueza (2020)	195	QUSF
CO38	*Cedrela odorata*	Galapagos Santa Cruz	−0.69937	−90.32312	G. Vinueza (2020)	184	QUSF
COISA12	*Cedrela odorata*	Galapagos Isabela	−0.84457	−91.02462	G. Vinueza (2020)	358	QUSF
COISA15	*Cedrela odorata*	Galapagos Isabela	−0.87375	−91.01208	G. Vinueza (2020)	178	QUSF
COISA13	*Cedrela odorata*	Galapagos Isabela	−0.85358	−91.01517	G. Vinueza (2020)	275	QUSF
COISA10	*Cedrela odorata*	Galapagos Isabela	−0.81240	−91.05247	G. Vinueza (2020)	541	QUSF
COISA11	*Cedrela odorata*	Galapagos Isabela	−0.83503	−91.03565	G. Vinueza (2020)	428	QUSF
COISA2	*Cedrela odorata*	Galapagos Isabela	−0.85333	−91.03600	G. Vinueza (2020)	390	QUSF
COISA8	*Cedrela odorata*	Galapagos Isabela	−0.83165	−91.05180	G. Vinueza (2020)	527	QUSF
COISA9	*Cedrela odorata*	Galapagos Isabela	−0.82163	−91.04710	G. Vinueza (2020)	498	QUSF
COISA1	*Cedrela odorata*	Galapagos Isabela	−0.86825	−91.01958	G. Vinueza (2020)	240	QUSF
COISA5	*Cedrela odorata*	Galapagos Isabela	−0.84545	−91.05072	G. Vinueza (2020)	489	QUSF
CO13	*Cedrela odorata*	Galapagos San Cristobal	−0.90027	−89.43775	G. Vinueza (2020)	237	QUSF
CO12	*Cedrela odorata*	Galapagos San Cristobal	−0.90035	−89.43792	G. Vinueza (2020)	238	QUSF
CO3	*Cedrela odorata*	Galapagos San Cristobal	−0.91095	−89.57610	G. Vinueza (2020)	192	QUSF
CO1	*Cedrela odorata*	Galapagos San Cristobal	−0.91092	−89.57597	G. Vinueza (2020)	193	QUSF
CO6	*Cedrela odorata*	Galapagos San Cristobal	−0.87702	−89.43752	G. Vinueza (2020)	324	QUSF
CO5	*Cedrela odorata*	Galapagos San Cristobal	−0.91132	−89.57570	G. Vinueza (2020)	187	QUSF
CO8	*Cedrela odorata*	Galapagos San Cristobal	−0.87693	−89.43775	G. Vinueza (2020)	326	QUSF
CO9	*Cedrela odorata*	Galapagos San Cristobal	−0.87690	−89.43777	G. Vinueza (2020)	327	QUSF
CO15	*Cedrela odorata*	Galapagos San Cristobal	−0.90005	−89.43725	G. Vinueza (2020)	232	QUSF
CO14	*Cedrela odorata*	Galapagos San Cristobal	−0.91597	−89.56182	G. Vinueza (2020)	238	QUSF
COFL13	*Cedrela odorata*	Galapagos Floreana	−1.31312	−90.47817	G. Vinueza (2020)	334	QUSF
COFL24	*Cedrela odorata*	Galapagos Floreana	−1.31372	−90.44008	G. Vinueza (2020)	326	QUSF
COFL2	*Cedrela odorata*	Galapagos Floreana	−1.32045	−90.44247	G. Vinueza (2020)	300	QUSF
COFL7	*Cedrela odorata*	Galapagos Floreana	−1.31770	−90.44653	G. Vinueza (2020)	326	QUSF
COFL11	*Cedrela odorata*	Galapagos Floreana	−1.31635	−90.44670	G. Vinueza (2020)	326	QUSF
COFL25	*Cedrela odorata*	Galapagos Floreana	−1.31382	−90.44875	G. Vinueza (2020)	346	QUSF
COFL3	*Cedrela odorata*	Galapagos Floreana	−1.31975	−90.44265	G. Vinueza (2020)	306	QUSF
COFL20	*Cedrela odorata*	Galapagos Floreana	−1.31542	−90.44492	G. Vinueza (2020)	333	QUSF
COFL17	*Cedrela odorata*	Galapagos Floreana	−1.31502	−90.44663	G. Vinueza (2020)	332	QUSF
COFL22	*Cedrela odorata*	Galapagos Floreana	−1.31567	−90.44273	G. Vinueza (2020)	312	QUSF
18332	*Cedrela “huberi”*	Ecuador Amazon	0.07380	−77.23960	P. Asadobay (2019)	389	QUSF
18350	*Cedrela “huberi”*	Ecuador Amazon	−0.79365	−76.88750	P. Asadobay (2019)	290	QUSF
18351	*Cedrela “huberi”*	Ecuador Amazon	−0.81336	−76.89020	P. Asadobay (2019)	299	QUSF
18352	*Cedrela “huberi”*	Ecuador Amazon	−0.83699	−76.89780	P. Asadobay (2019)	308	QUSF
18353	*Cedrela “huberi”*	Ecuador Amazon	−0.83826	−76.89800	P. Asadobay (2019)	309	QUSF
18354	*Cedrela “huberi”*	Ecuador Amazon	−0.94925	−76.91570	P. Asadobay (2019)	305	QUSF
18357	*Cedrela “huberi”*	Ecuador Amazon	−0.62820	−77.03770	P. Asadobay (2019)	321	QUSF
18358	*Cedrela “huberi”*	Ecuador Amazon	−0.70678	−77.08010	P. Asadobay (2019)	292	QUSF
18333	*Cedrela odorata*	Ecuador Amazon	0.06230	−77.29490	P. Asadobay (2019)	470	QUSF
18345	*Cedrela odorata*	Ecuador Amazon	−0.73057	−77.49470	P. Asadobay (2019)	688	QUSF
18347	*Cedrela odorata*	Ecuador Amazon	−0.47193	−76.98070	P. Asadobay (2019)	239	QUSF
18348	*Cedrela odorata*	Ecuador Amazon	−0.58117	−76.89100	P. Asadobay (2019)	258	QUSF
18349	*Cedrela odorata*	Ecuador Amazon	−0.65239	−76.88510	P. Asadobay (2019)	276	QUSF
18356	*Cedrela odorata*	Ecuador Amazon	−0.62365	−77.04200	P. Asadobay (2019)	333	QUSF
18359	*Cedrela odorata*	Ecuador Amazon	−0.89229	−77.18190	P. Asadobay (2019)	352	QUSF
18365	*Cedrela odorata*	Ecuador Amazon	−1.32650	−77.89230	P. Asadobay (2019)	1012	QUSF
18377	*Cedrela odorata*	Ecuador Amazon	−2.02000	−77.94720	P. Asadobay (2019)	975	QUSF
COT1	*Cedrela odorata*	Ecuador Amazon	−0.63898	−76.15513	P. Asadobay (2019)	215	QUSF
COT2	*Cedrela odorata*	Ecuador Amazon	−0.63897	−76.15512	P. Asadobay (2019)	211	QUSF
COT3	*Cedrela odorata*	Ecuador Amazon	−0.63907	−76.15505	P. Asadobay (2019)	210	QUSF
COT4	*Cedrela odorata*	Ecuador Amazon	−0.63490	−76.15497	P. Asadobay (2019)	208	QUSF
COT5	*Cedrela odorata*	Ecuador Amazon	−0.63472	−76.15537	P. Asadobay (2019)	229	QUSF
COT6	*Cedrela odorata*	Ecuador Amazon	−0.63467	−76.15538	P. Asadobay (2019)	230	QUSF
COT7	*Cedrela odorata*	Ecuador Amazon	−0.63475	−76.15532	P. Asadobay (2019)	231	QUSF
COT8	*Cedrela odorata*	Ecuador Amazon	−0.63475	−76.15532	P. Asadobay (2019)	230	QUSF
18425	*Cedrela odorata*	Ecuador Coast	0.62291	−79.90772	P. Asadobay (2019)	18	QUSF
18422	*Cedrela odorata*	Ecuador Coast	0.03584	−79.95113	P. Asadobay (2019)	261	QUSF
CC2	*Cedrela odorata*	Ecuador Coast	0.62294	−79.90767	G. Rivas‐Torres (2021)	20	QUSF
CC3	*Cedrela odorata*	Ecuador Coast	0.62294	−79.90767	G. Rivas‐Torres (2021)	20	QUSF
CC4	*Cedrela odorata*	Ecuador Coast	0.62294	−79.90767	G. Rivas‐Torres (2021)	20	QUSF
CC5	*Cedrela odorata*	Ecuador Coast	0.62286	−79.90766	G. Rivas‐Torres (2021)	19	QUSF
CC6	*Cedrela odorata*	Ecuador Coast	0.62287	−79.90764	G. Rivas‐Torres (2021)	18	QUSF
CC7	*Cedrela odorata*	Ecuador Coast	0.62285	−79.90766	G. Rivas‐Torres (2021)	17	QUSF
CC8	*Cedrela odorata*	Ecuador Coast	0.51069	−79.95727	G. Rivas‐Torres (2021)	10	QUSF
CC9	*Cedrela odorata*	Ecuador Coast	0.51063	−79.95728	G. Rivas‐Torres (2021)	9	QUSF
CC10	*Cedrela odorata*	Ecuador Coast	0.51055	−79.95734	G. Rivas‐Torres (2021)	9	QUSF
18423	*Cedrela brevicarpa* ^a^	Ecuador Coast	0.21922	−79.88104	P. Asadobay (2019)	10	QUSF
18424	*Cedrela brevicarpa* ^a^	Ecuador Coast	0.24578	−79.86882	P. Asadobay (2019)	6	QUSF
18426	*Cedrela brevicarpa* ^a^	Ecuador Coast	0.64255	−79.96195	P. Asadobay (2019)	18	QUSF
18428	*Cedrela brevicarpa* ^a^	Ecuador Coast	0.38845	−79.64418	P. Asadobay (2019)	333	QUSF
18429	*Cedrela brevicarpa* ^a^	Ecuador Coast	0.38845	−79.64418	P. Asadobay (2019)	333	QUSF
18453	*Cedrela brevicarpa* ^a^	Ecuador Coast	0.23878	−78.81811	P. Asadobay (2019)	693	QUSF
18454	*Cedrela brevicarpa* ^a^	Ecuador Coast	0.24031	−78.83042	P. Asadobay (2019)	575	QUSF
18742	*Cedrela brevicarpa* ^a^	Ecuador Coast	0.38777	−79.64082	P. Asadobay (2019)	426	QUSF
18743	*Cedrela brevicarpa* ^a^	Ecuador Coast	0.38777	−79.64082	P. Asadobay (2019)	426	QUSF
18397	*Cedrela angusticarpa*	Ecuador Andes	0.13740	−79.06990	P. Asadobay (2019)	505	QUSF
18406	*Cedrela angusticarpa*	Ecuador Andes	−0.02140	−78.88610	P. Asadobay (2019)	1191	QUSF
18407	*Cedrela angusticarpa*	Ecuador Andes	−0.04340	−78.96030	P. Asadobay (2019)	857	QUSF
18408	*Cedrela angusticarpa*	Ecuador Andes	−0.11930	−79.00430	P. Asadobay (2019)	698	QUSF
18411	*Cedrela angusticarpa*	Ecuador Andes	−0.17900	−79.03060	P. Asadobay (2019)	751	QUSF
18412	*Cedrela angusticarpa*	Ecuador Andes	−0.14955	−79.08610	P. Asadobay (2019)	581	QUSF
18413	*Cedrela angusticarpa*	Ecuador Andes	−0.14958	−79.08560	P. Asadobay (2019)	587	QUSF
18414	*Cedrela angusticarpa*	Ecuador Andes	−0.10248	−79.08448	P. Asadobay (2019)	614	QUSF
18445	*Cedrela angusticarpa*	Ecuador Andes	0.02583	−78.85944	P. Asadobay (2019)	1180	QUSF
18745	*Cedrela angusticarpa*	Ecuador Andes	0.34780	−79.67875	P. Asadobay (2019)	535	QUSF
18398	*Cedrela pubescens* ^a^	Ecuador Andes	0.15620	−78.81880	P. Asadobay (2019)	1578	QUSF
18399	*Cedrela pubescens* ^a^	Ecuador Andes	0.04600	−78.69950	P. Asadobay (2019)	1893	QUSF
18417	*Cedrela pubescens* ^a^	Ecuador Andes	−0.30854	−78.87741	P. Asadobay (2019)	1318	QUSF
18418	*Cedrela pubescens* ^a^	Ecuador Andes	−0.23104	−78.75646	P. Asadobay (2019)	1841	QUSF
18420	*Cedrela pubescens* ^a^	Ecuador Andes	−0.92958	−79.05002	P. Asadobay (2019)	1690	QUSF
18441	*Cedrela pubescens* ^a^	Ecuador Andes	−0.01333	−78.72056	P. Asadobay (2019)	1850	QUSF
18444	*Cedrela pubescens* ^a^	Ecuador Andes	0.03444	−78.72139	P. Asadobay (2019)	1720	QUSF
18455	*Cedrela pubescens* ^a^	Ecuador Andes	0.32571	−78.75055	P. Asadobay (2019)	1367	QUSF
18456	*Cedrela pubescens* ^a^	Ecuador Andes	0.33412	−78.74696	P. Asadobay (2019)	1426	QUSF
18457	*Cedrela pubescens* ^a^	Ecuador Andes	0.33290	−78.74834	P. Asadobay (2019)	1431	QUSF
18458	*Cedrela pubescens* ^a^	Ecuador Andes	0.32385	−78.75555	P. Asadobay (2019)	1380	QUSF
18459	*Cedrela pubescens* ^a^	Ecuador Andes	0.41580	−78.53699	P. Asadobay (2019)	1988	QUSF
18464	*Cedrela pubescens* ^a^	Ecuador Andes	0.45530	−78.52614	P. Asadobay (2019)	2107	QUSF
18466	*Cedrela pubescens* ^a^	Ecuador Andes	0.08709	−78.73502	P. Asadobay (2019)	1498	QUSF

^a^ W. Palacios et al., sp.nov.ined.

**TABLE A2:** Primer sequences and characterization of the nine microsatellite loci developed by Hernández et al. (2008).

Primer	Motive	Primer sequence (5′–3′)	Allele Size (bp)	Fluorophore‐labeled forward primers^a^
Ced2	(GA)_20_	F: TTTGCTTTGAGAAACCTTGT^a^ R: AACTTTCGAATTGGTTAAGG	130–170	PET
Ced18	(GA)_23_	F: CAAAGACCAAGATTTGATGC^a^ R: ACTATGGGTGGCACAACTAC	130–150	VIC
Ced131	(CT)_16_	F: CTCGTAATAATCCCATTCCA^a^ R:GGAGATATTTTTGGGGTTTT	80–120	6‐FAM
Ced65	(GA)_7_ (CA)_14_	F: GAGTGAGAAGAAGAATCGTGATAGC^a^ R: GAGGTTCGATCAGGTCTTGG	160–200	NED
Ced95	(CT)_17_ (AC)_13_	F: ATTTTCATTCCCTTTTAGCC^a^ R: TTATCATCTCCCTCACTCCA	190–250	6‐FAM
Ced44	(TG)_14_ (AG)_17_	F: ACTCCATTAACTGCCATGAA^a^ R: ATTTTCATTCCCTTTTAGCC	180–240	PET
Ced54	(GA)_15_(AG)_6_G(GA)_5_	F: GATCTCACCCACT TGAAAAA^a^ R: GCTCATATTTGAGAGGCATT	120–160	NED
Ced41	(TC)_18_	F: TCATTCTTGGATCCTGCTAT^a^ R: GTGGGAAAGATTGTGAAGAA	120–160	VIC
Ced61a	(TG)_10_	F: CAATCAAACCAAAAATGGAT^a^ R: GCAAATTAACCAGAAAAACG	240–270	6‐FAM

^a^ Position in primer where the fluorophore was attached.

**TABLE A3:** Final concentrations of BSA (mg/mL) and MgCl_2_ (Mm), annealing temperatures, and thermocycler cycles used in the amplification of nine microsatellite loci for *Cedrela odorata* in the Galapagos Islands and mainland Ecuador.

Primer	BSA (mg/mL)	MgCl_2_ (Mm)	Annealing temperature (°C)	Thermocycler cycles
Ced2	0.016	2.5	51^a^	30 (40^a^)
Ced18	1	2.5	55	35
Ced131	0.016	2.5	55	30
Ced65	0.016	2.5	55	30
Ced95	0.016	2.5	53^a^	35 (40^a^)
Ced44	0.016	2.5	51/53^a^	30 (40^a^)
Ced54	0.016	2.5	53^a^	30 (40^a^)
Ced41	0.016	2.5	53^a^	30 (40^a^)
Ced61a	1	3^a^	51	35–40

^a^ Modifications applied for only some Galapagos samples.

**TABLE A4:** Regions defined for samples of *Cedrela odorata* provided by Stephen Cavers used to perform Principal Coordinate Analyses (PcoA).

Region	Countries	Sampled individuals
North America	Mexico (Escarcega, Zona Maya, Guadalupe, unidentified)	36
Central America	Costa Rica	37
	Guatemala	25
	Honduras	14
	Nicaragua	4
	Panama	12
Caribbean	Cuba	174
South America	Brazil	31
	Colombia	5
	French Guyana	7
	Peru	51
	Ecuador	126

**TABLE A5:** Genetic diversity estimates for the nine microsatellite loci characterized for *Cedrela odorata* in the Galapagos Islands.

	*N*_a_	*H*_e_	*H*_o_	*N*_o_	*F*_ST_^A^	*F*_ST_^B^	HWD
Ced131	2	0.32	0.40	0	0.30	0.29	0.31
Ced18	3	0.49	0.07	0.28	−0.07	−0.05	0
Ced2	4	0.51	0.22	0.19	0.35	0.35	0
Ced65	2	0.48	0.40	0.06	0.29	0.29	0.33
Ced95	5	0.74	0.35	0.22	0.24	0.20	0
Ced44	4	0.70	0.42	0.16	0.36	0.33	0
Ced41	7	0.44	0.22	0.14	0.12	0.13	0
Ced54	3	0.51	0.48	0.02	−0.02	−0.02	0.61
Ced61a	5	0.74	0.65	0.06	0.13	0.12	0

Abbreviations: *F*_ST_^A^, uncorrected; *F*_ST_^B^, corrected for the effect of null alleles; *H*_e_, expected heterozygosity; *H*_o_, observed heterozygosity; HWD, deviation from Hardy Weinberg Equilibrium *p* value; *N*_a_, number of alleles; *N*_o_, null allele frequency.

**TABLE A6:** Genetic diversity parameters estimated for the islands where *Cedrela odorata* was sampled in the Galapagos Islands.

	*N*	*N*_a_	*H*_e_	*H*_o_	*F*_IS_	*N*_pa_	*R*_s_
Santa Cruz	10	29	0.55	0.53	0.07	5	3.15
Isabela	10	21	0.40	0.30	0.28	1	2.29
San Cristobal	10	22	0.35	0.23	0.36	2	2.32
Floreana	10	23	0.45	0.37	0.22	2	2.54

Abbreviations: *F*_IS_, inbreeding coefficient; *H*_e_, expected heterozygosity; *H*_o_, observed heterozygosity; *N*, number of individuals; *N*_a_, number of alleles; *N*_pa_, number of private alleles; *R*_s_, allelic richness.

**TABLE A7:** Demographic parameters obtained by DIYABC analysis of *Cedrela odorata* in Galapagos for the best supported scenario of Stage 1 (using only samples from Galapagos).

Parameter	Mean	Median	Mode	Quantile 2.5%	Quantile 5%	Quantile 25%	Quantile 75%	Quantile 95%	Quantile 97.5%	Relative median absolute errors
NE	2060	1370	86.1	56.5	108	564	2860	6610	7900	0.686
*N*2	6340	6580	7900	1000	1800	4750	8270	9650	9830	0.272
*N*3	5220	5280	8560	326	633	2850	7720	9560	9780	0.395
*N*4	4970	4810	3660	413	766	2660	7310	9460	9740	0.409
*N*5	4230	3700	2070	281	624	2090	6220	9110	9530	0.454
t1	779	610	414	97.7	147	366	997	2000	2430	0.402
t2	3240	3000	2440	1000	1200	2100	4140	6080	6700	0.303
t3	5000	4870	4760	1720	2050	3550	6390	8310	8730	0.250
t4	6280	6350	5740	2250	2670	4640	8060	9580	9800	0.184
μmic	1.36e‐004	1.24e‐004	1.00e‐004	1.00e‐004	1.00e‐004	1.10e‐004	1.49e‐004	2.08e‐004	2.37e‐004	0.244

*Note*: NE, *N*2, *N*3, *N*4 and *N*5 represent effective population sizes corresponding to the origin population, Santa Cruz, Isabela, San Cristobal and Floreana populations. Times of divergence between populations (*t*1, *t*2, *t*3, *t*4), the mean mutation rate of SSRs (μmic) and the relative median absolute error values based on 500 test datasets are also included.

**TABLE A8:** Demographic parameters obtained by DIYABC analysis of *Cedrela odorata* in Galapagos for the best supported scenario of Stage 2 (using samples of mainland Ecuador as the population of origin).

Parameter	Mean	Median	Mode	Quantile 2.5%	Quantile 5%	Quantile 25%	Quantile 75%	Quantile 95%	Quantile 97.5%	Relative median absolute errors
*N*1	9390	9540	9970	7920	8260	9140	9800	9970	9980	0.090
*N*2	2830	2000	1230	306	505	1190	3770	8060	8890	0.582
*N*3	4530	4250	1850	235	454	2020	6920	9350	9670	0.444
*N*4	2260	1180	310	121	186	494	3300	7910	8820	1.115
*N*5	4560	4220	1670	309	591	2100	6950	9360	9660	0.490
*t*1	6630	451	257	86.1	117	258	801	1920	2570	0.536
*t*2	162	115	70.6	27.6	35.7	71.7	192	442	596	0.441
*t*3	4870	4730	4150	1650	1980	3400	6230	8220	8680	0.265
*t*4	6390	6470	6840	2340	2810	4740	8150	9620	9820	0.169
μmic	6.32e‐004	6.41e‐004	6.81e‐004	2.63e‐004	3.22e‐004	5.09e‐004	7.65e‐004	9.08e‐004	9.44e‐004	0.168

*Note*: *N*1, *N*2, *N*3, *N*4 and *N*5 represent effective population sizes corresponding to the mainland Ecuador population, Santa Cruz, Isabela, San Cristobal and Floreana populations. Times of divergence between populations (*t*1, *t*2, *t*3, *t*4), the mean mutation rate of SSRs (μmic) and the relative median absolute error values based on 500 test datasets are also included.

**TABLE A9:** Demographic parameters obtained by DIYABC analysis of *Cedrela odorata* in Galapagos for the best supported scenario of Stage 3 (using the samples from the Coast of Ecuador as the population of origin).

Parameter	Mean	Median	Mode	Quantile 2.5%	Quantile 5%	Quantile 25%	Quantile 75%	Quantile 95%	Quantile 97.5%	Relative median absolute errors
*N*1	8270	8460	9000	5410	6030	7600	9140	9780	9880	0.173
*N*2	5140	5100	4500	423	839	3000	7350	9430	9710	0.331
*N*3	4430	4070	1950	231	467	2000	6720	9310	9620	0.446
*N*4	3670	2860	1730	590	805	1720	5230	8970	9500	0.452
*N*5	4640	4410	2720	245	477	2200	7020	9370	9690	0.407
*t*1	1390	1160	925	224	325	740	1790	3250	3910	0.412
*t*2	1790	1550	1180	477	583	1050	2280	3780	4410	0.413
*t*3	3230	2920	2150	993	1180	2040	4140	6310	7140	0.208
*t*4	7720	8010	9770	4040	4670	6660	9050	9810	9910	0.144
μmic	1.98e‐004	1.76e‐004	1.35e‐004	1.06e‐004	1.11e‐004	1.39e‐004	2.31e‐004	3.62e‐004	4.26e‐004	0.288

*Note*: *N*1, *N*2, *N*3, *N*4 and *N*5 represent effective population sizes corresponding to the Coast population, Santa Cruz, Isabela, San Cristobal and Floreana populations. Times of divergence between populations (*t*1, *t*2, *t*3, *t*4), the mean mutation rate of SSRs (μmic) and the relative median absolute error values based on 500 test datasets are also included.
